# Tumor characterization by ultrasound-release of multiple protein and microRNA biomarkers, preclinical and clinical evidence

**DOI:** 10.1371/journal.pone.0194268

**Published:** 2018-03-16

**Authors:** Aloma L. D’Souza, John R. Chevillet, Pejman Ghanouni, Xinrui Yan, Muneesh Tewari, Sanjiv S. Gambhir

**Affiliations:** 1 Departments of Radiology, Stanford University, Stanford, California, United States of America; 2 Molecular Imaging Program, Stanford University, Stanford, California, United States of America; 3 Division of Human Biology, Fred Hutchinson Cancer Research Center, Seattle, Washington, United States of America; 4 Bioengineering, Stanford University, Stanford, California, United States of America; Columbia University, UNITED STATES

## Abstract

We have previously shown that low frequency ultrasound can release biomarkers from cells into the murine circulation enabling an amplification and localization of the released biomarker that could be used as a blood-based method to detect cancer earlier and monitor therapy. In this study, we further demonstrate that this technique could be used for characterization of tumors and/or identification of cellular masses of unknown origin due to the release of multiple protein and nucleic acid biomarkers in cells in culture, mice and patients. We sonicated colon (LS174T) and prostate (LNCaP) cancer cell lines in culture at a low frequency of 1 MHz and show that there were several-fold changes in multiple protein and microRNA (miRNA) abundance with treatment at various intensities and time. This release was dependent on the duration and intensity of the sonication for both cell lines. Significant increased release in biomarkers was also observed following tumor sonication in living mice bearing subcutaneous LS174T cell line xenografts (for proteins and nucleic acids) and in an experimental LS174T liver tumor model (for proteins only). Finally, we demonstrated this methodology of multiple biomarker release in patients undergoing ablation of uterine fibroids using MR guided high intensity focused ultrasound. Two protein biomarkers significantly increased in the plasma after the ultrasound treatment in 21 samples tested. This proof that ultrasound-amplification method works in soft tissue tumor models together with biomarker multiplexing, could allow for an effective non-invasive method for identification, characterization and localization of incidental lesions, cancer and other disease. Pre-treatment quantification of the biomarkers, allows for individualization of quantitative comparisons. This individualization of normal marker levels in this method allows for specificity of the biomarker-increase to each patient, tumor or organ being studied.

## Introduction

Non-invasive detection of circulating biomarkers can potentially revolutionize clinical medicine by earlier cancer diagnosis and monitoring of cancer therapy [1, 2]. Three fundamental problems impeding blood-based cancer biomarker research are: 1) low blood biomarkers concentrations that cannot be differentiated from normal background variations; 2) the presence of a blood-based cancer biomarker yields no information about the location of the biomarker production site(s); and 3) high variability of biomarker levels within a healthy patient population makes it difficult to standardize a cutoff concentration for disease [3]. Ultrasound has been used for intracellular delivery of molecules into cells because of its property of reversibly compromising the cell membrane due to cavitation at frequencies from 20 kHz to 16 MHz with very little cellular death [4, 5]. We previously demonstrated that this bioeffect of ultrasound at 1 MHz can amplify the release into circulation and localize the production site of a biomarker from a colon cancer cell line in culture and in a subcutaneous tumor model in living mice [6]. We hypothesized and systematically proved that ultrasound can cause the release of the biomarker in a spatially localized manner, only when it is applied to the cells producing the specific biomarker. Each mouse served as its own internal control before and after sonication of the tumor mass, which helps in individualization of the relative changes in biomarker levels. The study demonstrated that ultrasonic energy deposited on macroscopic tumors could potentially overcome the major current limitations of serum biomarkers mentioned above.

Magnetic resonance guided-high intensity focused ultrasound (MRg-FUS) is used clinically to ablate uterine fibroids and bone metastasis [7, 8] and is being developed for other clinical applications, including prostate, breast and soft tissue tumors [9–12]. Magnetic resonance (MR) imaging is used to visualize the target for localized ultrasound energy deposition, to measure the temperature at the target during treatment and to assess immediately post-operatively the percentage of the targeted volume that has been ablated. Uterine fibroids are common benign tumors that with growth in size can give rise to unbearable symptoms and MRg-FUS is rapidly gaining favor as a non-invasive treatment of uterine fibroids. Ablation of the fibroid due to an increase in temperature causes cellular destruction, necrosis and coagulation [13]. We have shown that an increase in cell death increases biomarker-release [6] and therefore the use of MRg-FUS for ablation could allow for higher amplification of biomarker levels in humans, where the dilution of the biomarker in a large blood volume may limit detection. Recently, a systematic preclinical study from the authors has shown that both cavitation and liquefaction after pulsed focused sonication of tumors in rats can cause an increase in microRNA (miRNA) levels in plasma [14]. MRg-FUS has been shown to be safe in the use of tumor eradication and control of tumor growth by an increase in anti-tumor immune responses in several clinical and preclinical studies ([10, 14]). The study of the use of MRg-FUS for the release of biomarkers from the benign human fibroids will guide us in the adaptation of the system for use in tumor biomarker release and localization at lower non-ablating ultrasound depositions.

In the clinical environment of blood-based biomarker studies it has been shown that a single biomarker is generally not effective in detecting or guiding management and detection of disease, and that panels of multiple biomarkers are required [15–19]. Protein blood biomarkers such as carcinoembryonic antigen (CEA), cancer antigen 19–9 (CA19-9) and prostate specific antigen (PSA) used in this study, have been widely studied for many decades and are used in mainstream clinical disease management [20–24]. Endothelin-1, cancer antigen 125 (CA125) and cancer antigen 15–3 (CA15-3) have been studied in uterine fibroids [25–27]. Circulating nucleic acid biomarkers are also being explored as potential non-invasive disease detection agents. MicroRNAs have been shown to circulate in a blood in a highly stable and cell-free form and profiles of miRNAs have been shown to be distinguishable between cancer and other disease [28–31]. The long half-life of miRNA in plasma and the amplification of miRNA in its analysis allows for an increased analytical sensitivity in detection compared to protein biomarkers.

In this study, we demonstrate that ultrasound amplifies the release of multiple proteins and miRNAs from multiple cell lines in culture, in living mice and in patients. We use 1 MHz sonication at a low intensity to perturb the cell lines and show that there is significant release of biomarkers into the extracellular milieu. We were able to demonstrate the ultrasound induced biomarker release in a liver xenograft tumor model, which is more generalizable to a clinically relevant scenario of soft tissue tumor growth. Also for the first time we were able to study an extension of this effect in patients being treated for uterine fibroid ablation with MRg-FUS at 1.1 MHz. This study extends our initial report allowing for a more concrete evidence that ultrasound can allow for a more accurate and noninvasive clinical strategy for characterization and spatial localization of disease.

## Materials and methods

### Cell culture

Cell lines were obtained from American Type Culture Collection (ATCC). The colon cancer cell line LS174T was cultured in DMEM:F12 nutrient mix complete media (Gibco 11330) and the prostate cancer cell line LNCaP was cultured in RPMI-1640 medium (ATCC 30–2001). Media was supplemented with 10% fetal bovine serum (Gibco 26140).

### Protein quantification

Samples of culture media or plasma were analyzed for concentrations of CEA, CA19-9 or PSA using ELISA kits from United Biotech, Inc. (CEA: CM-201; CA19-9: CM-801), R&D systems, Inc. (PSA: DKK300; endothelin-1: DET100; CA125: DCA125) and RayBiotech, Inc. (CA15-3: ELH-CA15-3). The standards of the kits used for the mice plasma were modified by dilution of the lowest standard to allow for a more reliable curve fit at the lower expected concentrations. The sensitivity of the assays were: CEA-0.05 ng/ml, CA19-9-0.03 U/ml, PSA-0.2 ng/ml, endothelin-1-0.031 pg/ml, CA125-0.007 U/ml and CA15-3-4 mU/ml.

### MicroRNA quantification

miRNA was determined in cell culture media samples and mice and human plasma samples using TaqMan reverse-transcription quantitative polymerase chain reaction (RT-qPCR). The supernatant samples were collected and centrifuged for 10 min at 1,600 g at 4°C to remove any debris. The supernatant was further centrifuged at 16,000 g for 10 min at 4°C and aspirated to a clean tube. Plasma samples were collected in EDTA-tubes and plasma was separated by centrifugation at 1000g, at 4°C for 10 min. An aliquot of the resulting supernatant and plasma was treated with 10 volumes of Qiazol (Qiagen Inc.; 79306). 5 μl of *C*.*elegans* spike-in control miRNAs were added before extraction of the total RNA by miRNAeasy RNA isolation kit (Qiagen Inc.) and analysis of the RNA by RT-qPCR as previously described [29–31]. RQ is relative quantity as calculated between pre and post values, using the efficiencies empirically determined from standard curves and the Pfaffl method [32]. The results were standardized to the spiked in *C*.*elegans* miRNAs.

### Ultrasound treatment of cells

The cells were plated in a well of a 6-well tissue culture plate (BD Biosciences: 35–3046) at a concentration of 1.5x10^6^ cells per well. The cells were incubated overnight in complete media and the next day the confluent cell layer was rinsed with PBS and fresh media containing protease inhibitors (Roche: 04693159001) was added to the cells just before the start of sonication. The cells were sonicated using a 20 mm transducer of the Sonitron 2000 (Artison Corp.) from the bottom of the plate. Ultrasound-coupling gel (Aquasonic 100; Parker Laboratories, Inc.) was used for coupling the plate to the transducer. Media samples were collected before sonication and at the indicated time points, then centrifuged to remove any debris and analyzed for the various biomarkers. Control samples were treated without ultrasound for the same duration of time as the sonicated samples.

### Ultrasound treatment of mouse xenograft models

Mice were kept under anesthesia (2% vol/vol isofluorane in oxygen) for all procedures and were carefully monitored and all efforts were made to minimize any distress in the mice. Eight-week old female nude mice (nu/nu; Charles Rivers Laboratories, Inc.) were inoculated subcutaneously on the right flank with 3x10^6^ LS174T cells suspended in 0.1 ml phosphate buffered saline, using a 28-gauge 0.5-inch needle. The tumors were allowed to grow for 2–3 weeks. For the liver tumor transplantation, a 2–3 mm^3^ piece of the above subcutaneous tumor, after a week of growth, was implanted into a small incision in the left liver lobe after exposure of the liver by laparotomy. The incision was sutured and the tumor growth was followed using ultrasound imaging using the Visual Sonic Vevo 2100. The tumors were allowed to grow for approximately 2 weeks till most of the tumors reached a minimum volume of 0.3 cm^3^ (Volume: Average = 0.8 ± 0.5 cm^3^; Minimum = 0.3 cm^3^; Maximum = 1.7 cm^3^). For treatment of the tumors the mice were anaesthetized with 2% (vol/vol) isofluorane in oxygen and kept warm on a heated pad during the treatment. Pre-ultrasound samples were collected by an approximately 75 μl submandibular bleed before sonication. The skin surrounding the tumor was cleaned with an alcohol wipe and ultrasound-coupling gel (Aquasonic 100) was then layered on the tumor and the 20 mm transducer of the Sonitron 2000 was placed directly over the known tumor or area of the tumor. The tumor was sonicated for the indicated times and a submandibular blood sample was collected and separated into plasma (BD Biosciences: 36–5958 for protein and 36–5973 for miRNA). The volume of blood per collection was approximately 75 μl. 200 μl of warm saline was subcutaneously administered to compensate for the loss of fluid. Control mice were treated under the same conditions and for the same duration of time without sonication. Animals were monitored every 2–3 days during tumor growth for any signs of distress, lethargy, difficulty breathing, dehydration, skin laceration or weight loss. Animals were euthanized with carbon dioxide inhalation at the end of the above detailed experimental procedures or if early euthanasia was required. The animal protocol was carried out in strict accordance with the approval of the Stanford Administrative Panel on Laboratory Animal Care. All animal procedures and imaging were carried out in the Stanford Small Animal Imaging Facility.

### MRg-FUS treatment of uterine fibroids in patients

Patients, who are routinely treated for uterine fibroids by ablation by MRg-FUS at Stanford Fibroid Center at the Stanford Hospital and Clinics, were recruited for this trial. Patients are clinically treated using the ExAblate 2100 system (InSightec, Israel) [7, 8], at a frequency of 1.1 MHz and power of 100–200 W. The ablation causes a temperature rise of about 20–30°C and the spot size used varied from 4.5–7.5 mm in diameter to 14.5–70 mm in length. Blood was collected from the patients before and as soon as possible after fibroid treatment (within 30 min) and immediately processed for plasma separation. Plasma samples were collected in EDTA-tubes (BD 367899) and plasma was separated by centrifugation at 1000g, at 4°C for 10 min. The plasma was used to detect biomarkers that could be released into it due to the focused ultrasound treatment. All patients provided informed written consent and the protocol was carried out in strict accordance with the approval of the Administrative Panel on Human Subjects in Medical Research and Stanford Institutional Review Board at Stanford University.

### Statistical analysis

For analysis of the cell culture experiments of progressive release of biomarkers under ultrasound treated and control conditions statistical significance was determined using unpaired t-test with correction for multiple comparisons by the Holm-Sidak method. For multiple comparisons between collection times and treatment conditions, 2-way Analysis of Variance (ANOVA) was used. For the living mice studies, unpaired one-tailed t-test was done with Welch’s correction was done unless mentioned otherwise in the results section. Outliers were detected using ROUT (Q = 1–5%). For the human studies, Wilcoxon two-tailed paired test were done on the pre- and post-treatment values and linear and non-linear quadratic regression analysis was done for the correlation studies. A p-value ≤0.05 was considered to be significant. Analysis was done using GraphPad Prism 6.0h.

## Results

### Cell culture studies

#### Release of biomarkers from the colon cancer cell line LS174T in culture

**Fig 1:**
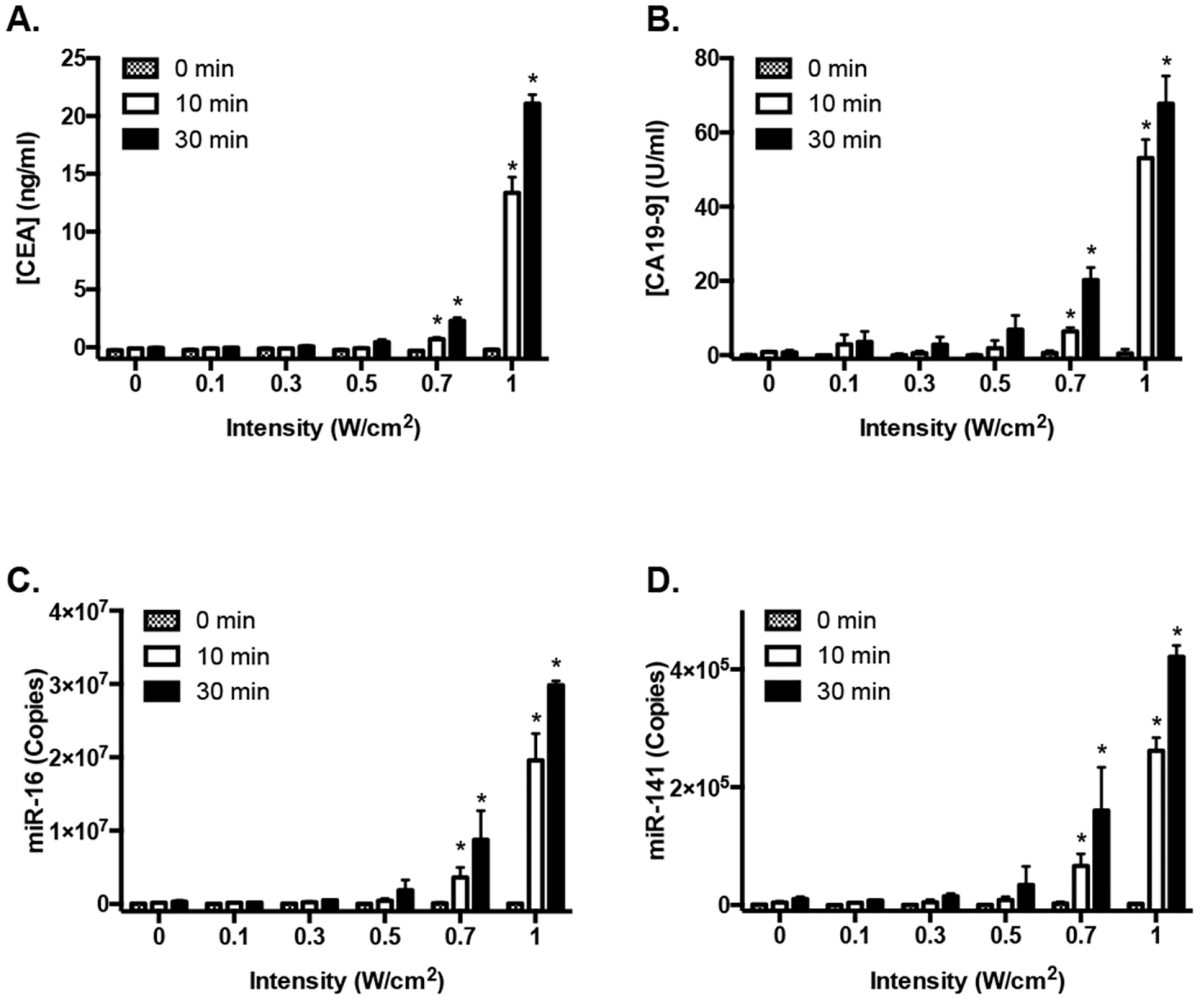
Release of biomarkers from LS174T cells in culture treated with varying ultrasound intensities. Cells in culture (n = 2 wells) were exposed to varying ultrasound intensities (0, 0.1, 0.3, 0.5, 0.7 and 1.0 W/cm^2^) at 1 MHz and 10% duty cycle. The biomarker levels were studied in the media and were seen to progressively increase with an increase in intensity as well as an increase in time (0, 10, 30 min). There was a significant (*p≤0.05) increase in CEA (**A**), CA19-9 (**B**), miR-16 (**C**) and miR-141 (**D**) at 0.7 and 1 W/cm^2^ for 10 and 30 min of treatment. Error bars are SD.

In the current study, ultrasound-dependent amplification of biomarkers from the colon cancer cell line LS174T, was studied. Cells were treated with varying ultrasound intensities (0, 0.1, 0.3, 0.5, 0.7, 1.0 W/cm^2^) at 1 MHz and 10% duty cycle for 30 min (n = 2). Intensity is defined as the power (W) divided by the area (cm^2^) and duty cycle is the percentage of time the sound wave is on within a cycle. The media was collected at times 0, 10 and 30 min and analyzed. The protein biomarkers CEA and CA19-9 were found to steadily increase with both an increase in intensity and time of ultrasound treatment (Fig 1A and 1B). For CEA significance was reached for 0.7 W/cm^2^ (10 min: 2-fold increase, p = 0.039; 30 min: 8-fold increase, p<0.0001) and for 1 W/cm^2^ (10 min: 63-fold increase, p<0.0001, 30 min: 100-fold increase, p<0.0001) (Fig 1A). For CA19-9 significant results were obtained for 0.7 W/cm^2^ (30 min: 35-fold increase, p<0.0001) and for 1 W/cm^2^ (10 min: 122-fold increase, p<0.0001; 30 min: 155-fold increase, p<0.0001) (Fig 1B).

MicroRNAs, miR-16 and miR-141, were also analyzed. miR-16 is a widely expressed miRNA across cell types, whereas miR-141 is associated with epithelial cells [31, 33, 34]. MicroRNA data was expressed the number of copies detected. Ultrasound treatment increased the abundance of cell-free miRNAs released into the media, also in an intensity and time dependent manner. This release was seen to increase relative to pre-treatment baseline (0-min) levels, from 12-fold (0.1 W/cm^2^) to 568-fold (1.0 W/cm^2^) for miR-16 and 60-fold (0.1 W/cm^2^) to 212-fold (1.0 W/cm^2^) for miR-141, following 30 min of treatment (Fig 1C and 1D). The data was significant within 10 min for 0.7 and 1 W/cm^2^ for miR-16 (0.7 W/cm^2^: 10 min: p = 0.04; 30 min: p<0.0001; 1 W/cm^2^: 10 min: p<0.0001, 30 min: p<0.0001) and miR-141 (0.7 W/cm^2^: 10 min: p = 0.017, 30 min: p<0.0001; 1 W/cm^2^: 10 min: p<0.0001, 30 min: p<0.0001).

**Fig 2:**
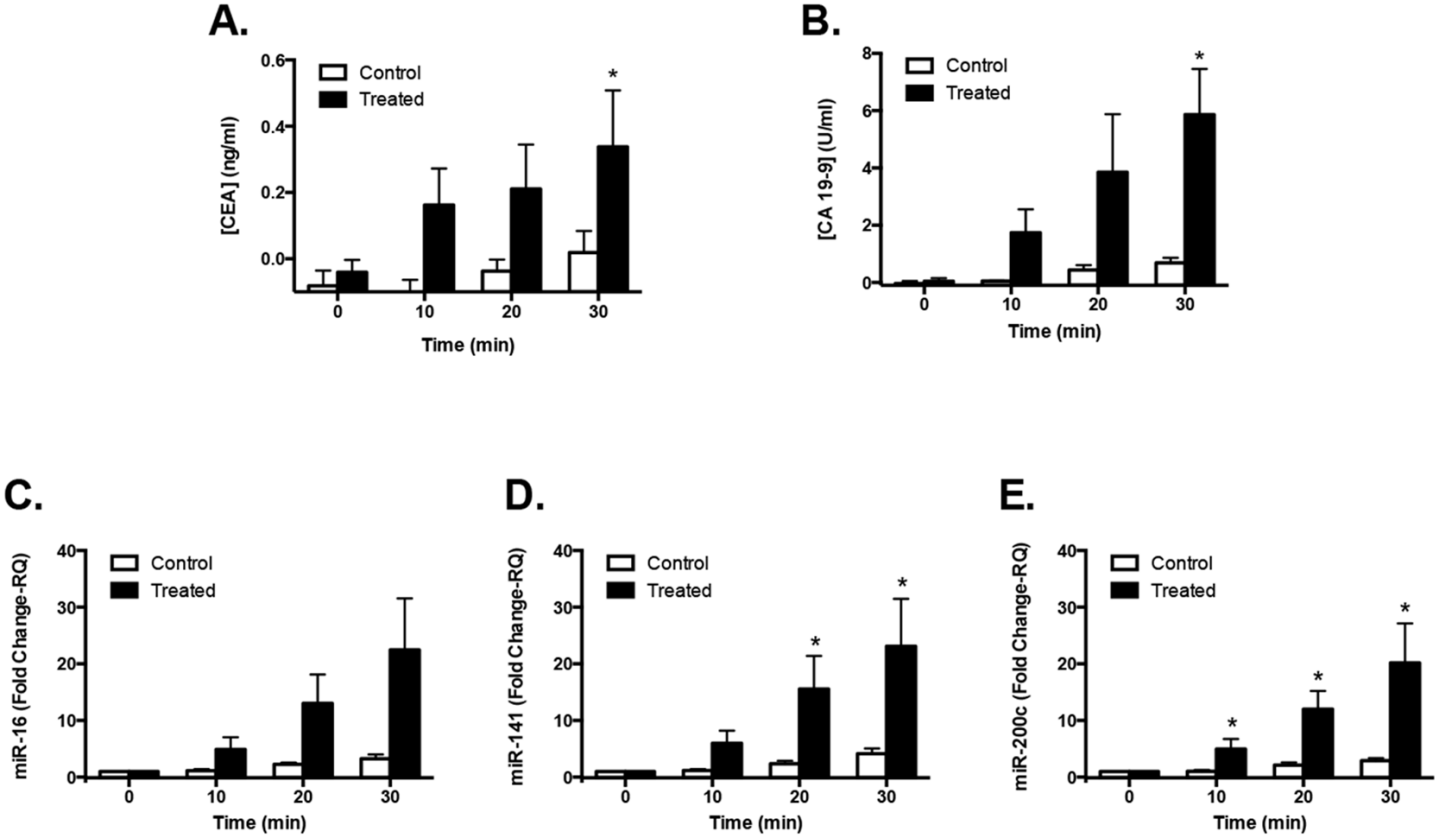
Time release of biomarkers from LS174T cells in culture treated with 0.5 W/cm^2^ ultrasound intensity. Cells (n = 3 wells) were exposed to a constant ultrasonic pulse (1MHz, 0.5 W/cm^2^, 10% duty cycle) for 30 min. All the biomarkers showed a significant (p≤0.05) increase at 30 min post-treatment with ultrasound compared to the pre-treatment time point: CEA (**A**), CA19-9 (**B**), miR-16 (**C**), miR-141 (**D**) and miR-200c (**E**). Untreated controls did not show significant increases. Increase of ultrasound-treated to untreated-controls at the same time point reached significance for some of the biomarkers (*p≤0.05). Error bars are SEM. RQ is relative quotient indicative of fold change.

Sonication of the cells was also studied with a higher number of replicates (n = 3) at 0.5 W/cm^2^, 10% duty cycle at 1 MHz with media collection at 0, 10, 20 and 30 min. This was done as we have shown that there is little cell death at this intensity [6]. Thirty minutes after ultrasound treatment, levels for both protein biomarkers were significantly increased when compared to the 0-min time point (CEA: 8-fold increase, p = 0.006; CA19-9: 135-fold increase, p = 0.0001) and this significance was not reached for the control group (CEA: 0-fold increase, p = 0.85; CA19-9: 19-fold increase, p = 0.83) (Fig 2A and 2B). Both protein biomarker levels in the media were significantly higher at 30 min when compared to the untreated controls at the same time-point (CEA: 18-fold increase, p = 0.03; CA19-9: 9-fold increase, p = 0.03). MicroRNAs, expressed as relative quotient (RQ) indicative of fold change (Fig 2C, 2D and 2E), also showed significant increases at 30 min by when compared to baseline (0 min) for all the three miRNAs studied (miR-16: 23-fold increase, p = 0.0001; miR-141: 23-fold increase, p<0.0001; miR-200c: 20-fold increase, p<0.0001) and whereas there was no significant change in miRNA levels for the control group within the same time (miR-16: 3-fold increase, p = 0.99; miR-141: 4-fold increase, p<0.86; miR-200c: 3-fold increase, p<0.93). The three miRNAs trended towards or reached significant increases in release at 30 min when compared to the untreated controls at the same time-point (miR-16: 7-fold increase, p = 0.06; miR-141: 6-fold increase, p = 0.05; miR-200c: 7-fold increase, p = 0.03). Significance at earlier time points (10 and/or 20 min) when compared to controls at those same time points were reached for some of the markers (miR-16: 10 min: p = 0.1, 20 min: p = 0.06; miR-141: 10 min: p = 0.6, 20 min: p = 0.05; miR-200c: 10 min: p = 0.05, 20 min: p = 0.01).

#### Release of biomarkers from the prostate cancer cell line LNCaP in culture

**Fig 3:**
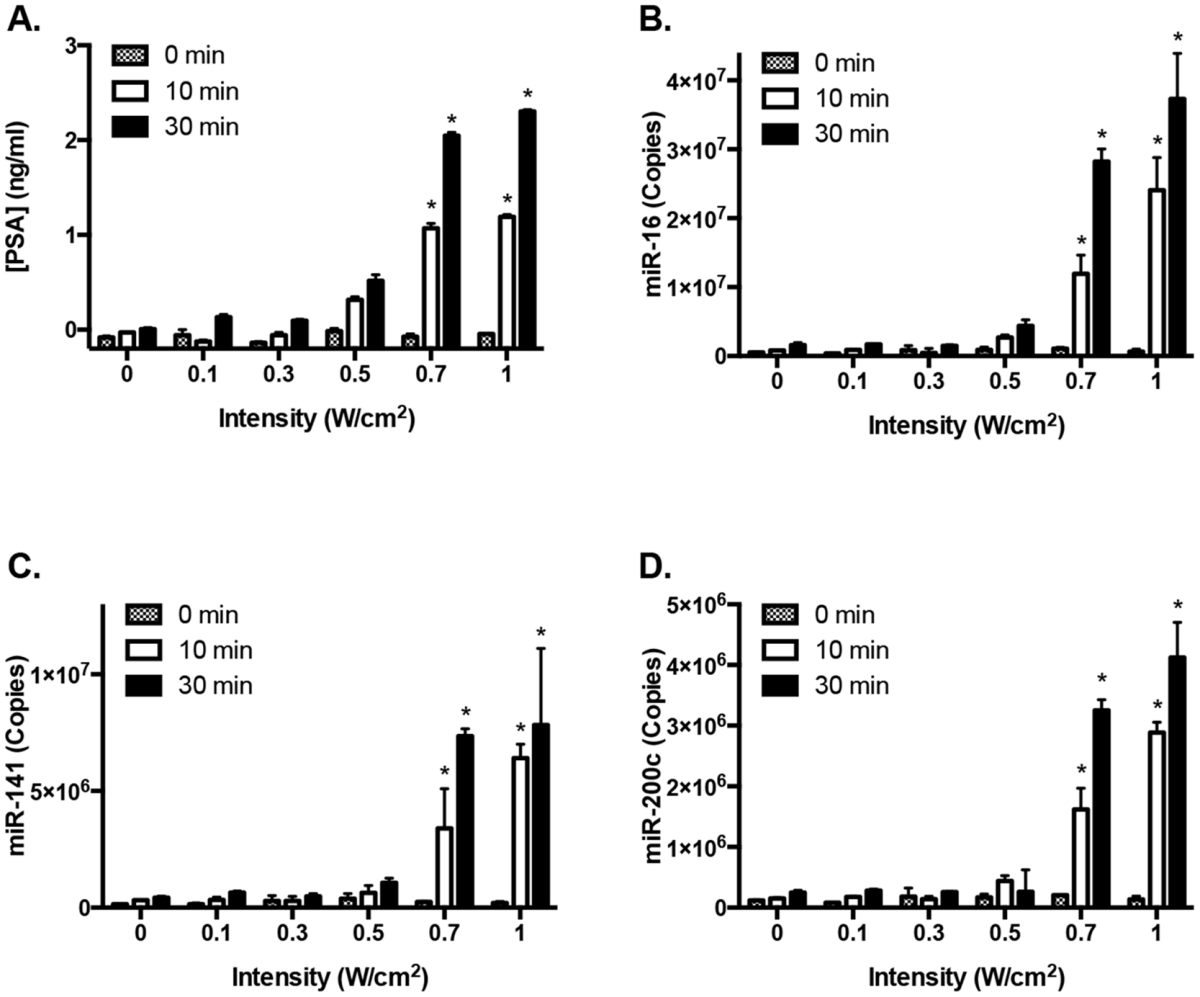
Release of biomarkers from LNCaP cells in culture with varying ultrasound intensities. To study the effects of ultrasound on the release of biomarkers from a different cell line, LNCaP cells (n = 2 wells) were treated in culture with varying ultrasound intensities (0, 0.1, 0.3, 0.5, 0.7 and 1.0 W/cm^2^) at 1 MHz frequency and 10% duty cycle. All the biomarkers showed an increase in release with increase in ultrasound intensity with time (0, 10, 30 min). There was a significant (*p≤0.05) increase in PSA (**A**), miR-16 (**B**), miR-141 (**C**) and miR-200c (**D**) at 0.7 and 1 W/cm^2^ for 10 and 30 min of treatment. Error bars are SD.

Ultrasound effects on the biomarker release from the prostate epithelial cancer cell line LNCaP were also studied. Prostate specific antigen (PSA) is a well-established biomarker that is expressed and released from LNCaP cells [35]. miR-16 the epithelial marker, expressed in these cells [36] together with widely expressed nucleotide markers, miR-141 and miR-200c were studied. The cells were studied at the varying ultrasound intensities as was done for the LS174T cell line. Media was collected at 0, 10, 30 min was analyzed for PSA, miR-16, miR-141 and miR-200c (n = 2). PSA showed a progressive increase in released levels with an increase in ultrasound treatment time (Fig 3A). This reached significance for 0.7 (10 min: 15-fold increase, p = 0.001; 30 min: 28-fold increase, p<0.0001) and 1 (10 min: 27-fold increase, p = 0.0003; 30 min: 53-fold increase, p<0.0001) W/cm^2^. Untreated control samples showed a very slight increase in PSA after 30 min (0.09-fold for 0 W/cm^2^). A significant increase was also observed with all the miRNA levels by 10 min for 0.7 (miR-16: 10 min: 11-fold increase, p = 0.0002, 30 min: 26-fold increase, p<0.0001; miR-141: 10 min: 13-fold increase, p = 0.007, 30 min: 30-fold increase, p<0.0001; miR-200c: 10 min: 8-fold increase, p<0.0001, 30 min: 15-fold increase, p<0.0001) and 1 W/cm^2^ (miR-16: 10 min: 38-fold increase, p<0.0001, 30 min: 59-fold increase, p<0.0001; miR-141: 10 min: 32-fold increase, p<0.0001, 30 min: 39-fold increase, p<0.0001; miR-200c: 10 min: 21-fold increase, p<0.0001, 30 min: 31-fold increase, p<0.0001) (Fig 3B, 3C and 3D). The untreated controls (0 W/cm^2^) showed a <3-fold increase from 0–30 min for the miRNA levels.

### Living mice studies

#### Release of biomarkers from LS174T subcutaneous xenograft tumors in living mice

To demonstrate the amplified release of multiple biomarkers from tumors in living mice, LS174T cells were injected subcutaneously into the right flank of nude mice and grown into tumors. When the resulting tumors exceeded a volume of 0.3 cm^3^ (average 0.85 cm^3^) they were then treated with ultrasound (1 MHz at 2 W/cm^2^ at 50% duty cycle for 6 min as previously studied [6]. Control tumors were not sonicated and all other conditions were kept similar to the treated group. We have demonstrated that these ultrasound conditions caused the highest increase of release of CEA into the blood of living mice with minimum cell death [6]. Plasma was collected immediately after sonication for the protein biomarkers but within an hour for the miRNAs. The clearance of CEA from the blood in nude mice has been previously studied and shows a clearance of approximately 90% within 10 min, 95% by 30 min and 98% by 2 hours [37] and the half-life for CA19-9 is 6.5 h +/- 1.5 h [38]. MicroRNA are known to have long half-lives of >24 h, *ex vivo* [31].

**Fig 4:**
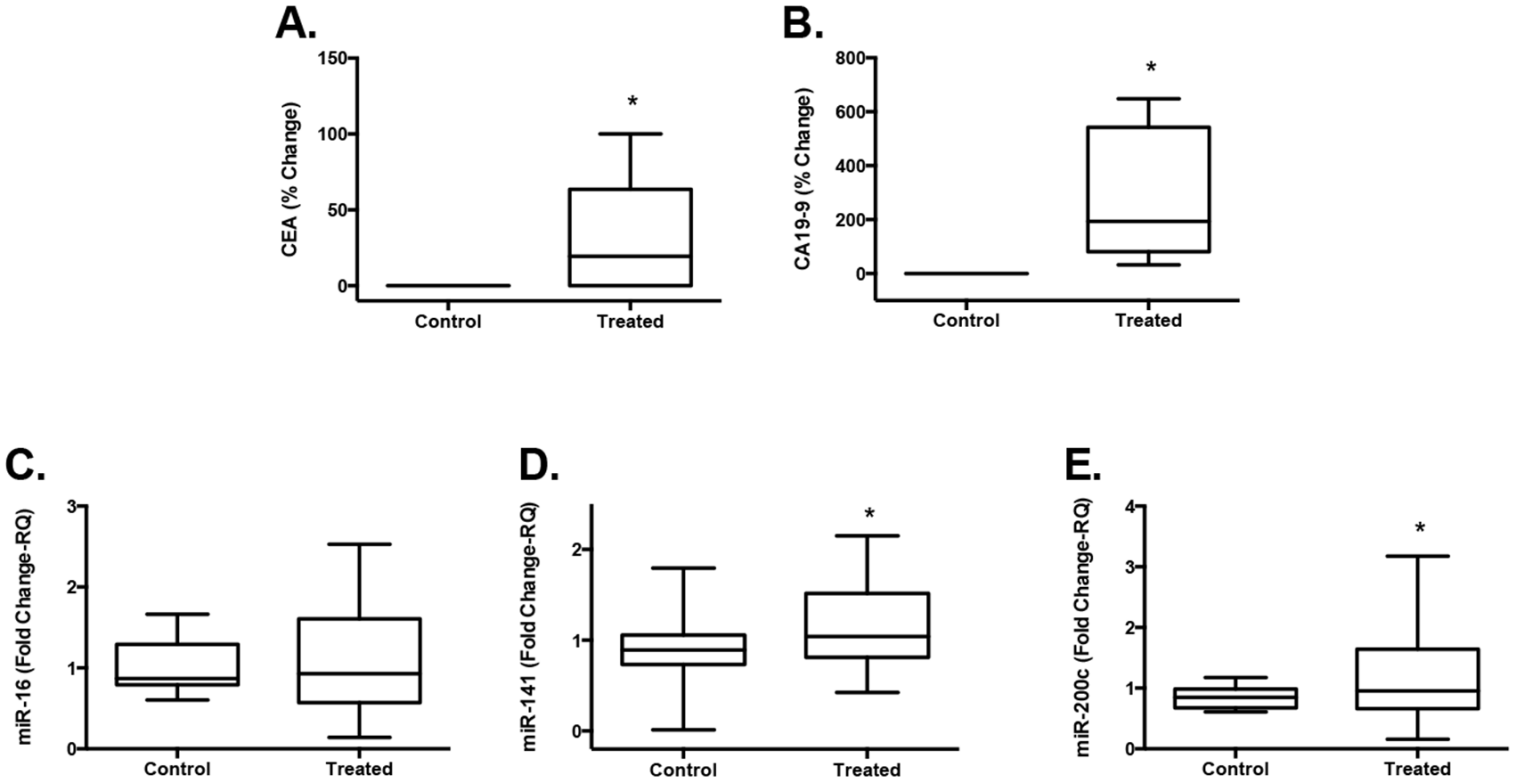
Release of biomarkers from LS174T subcutaneous tumors in living mice. Subcutaneous xenograft tumors of LS174T cells were grown in nude mice and tumors were treated with 1 MHz ultrasound at 2 W/cm^2^, 50% duty cycle for 6 min. Plasma was collected pre- and post-ultrasound treatment and analyzed for protein biomarkers (n = 10) and miRNAs (n = 21). The percent change between pre- and post-ultrasound levels in treated samples compared to untreated controls was significant (*p≤0.05) for CEA (**A**) and CA19-9 (**B**), miR-141 (**D**) and miR-200c (**E**) and not for miR-16 (p = 0.3) (**C**). Box of the plots indicate 25^th^ to 75^th^ percentile with the median and whiskers are maximum to minimum values.

The protein biomarkers exhibited a significant percent increase between post- and pre- ultrasound treatment levels in plasma in the treated mice (n = 10) (CEA: 33-fold increase, p = 0.02; CA19-9: 283-fold increase, p = 0.002) when compared to the untreated control (n = 5) levels (Fig 4A and 4B). MicroRNA levels were also higher after ultrasound treatment in the plasma of the treated mice (n = 21). The levels reached significance when comparing the treated mice to the untreated controls for miR-141 (p = 0.035) and miR-200c (p = 0.027) and did not reach significance for miR-16 (p = 0.3), even though it showed an increase over the controls (Fig 4C, 4D and 4E).

#### Release of biomarkers from LS174T liver xenograft tumors in living mice

**Fig 5:**
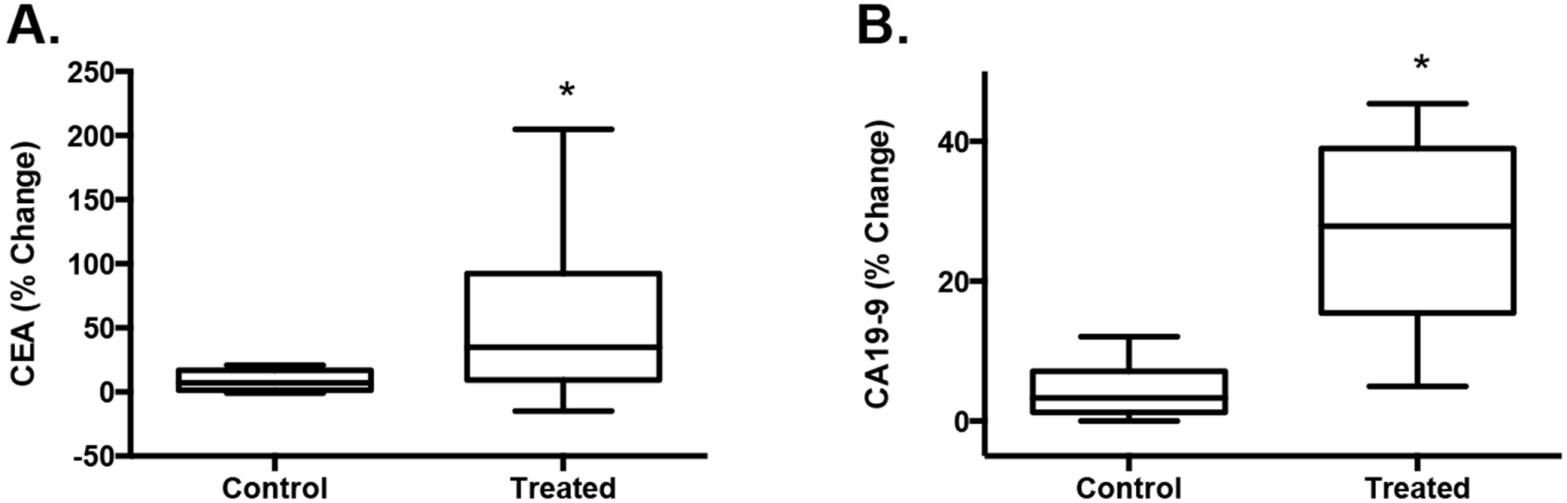
Release of biomarkers from LS174T liver tumors in living mice. LS174T tumors were grown within the liver of nude mice and treated with ultrasound at 1 MHz at 2 W/cm2, 50% duty cycle for 6 min (n = 10). Plasma collected pre- and post-sonication was collected and analyzed. The percent change in levels pre- and post-treatment showed significance (*p≤0.05) for CEA (**A**) and CA19-9 (**B**). Box of the plots indicate 25^th^ to 75^th^ percentile with the median and whiskers are maximum to minimum values.

Biomarkers released from a subcutaneous tumor may not be generalizable to tumors found within organs due to the differences in the tumor microenvironment and vasculature. To demonstrate the release of biomarkers from a tumor within an organ, a 2–3 mm^3^ piece of LS174T subcutaneous tumor was transplanted into the liver of living mice where it was allowed to grow [39]. This orthotopic model was chosen because of the ease of access to the liver for the ultrasound treatment, enabling us to study effects of sonication of cells in an experimental orthotopic soft tissue model (n = 10). The tumor size was followed using ultrasound imaging and sonication was done over the area of the tumor implantation. The tumors were allowed to reach at least 0.3 cm^3^ before sonication with the average volume being 0.8 cm^3^. The 20 mm transducer used has a well-collimated energy beam about 11 mm across at a depth of 10–15 mm, ensuring ample coverage of the tumor within the liver of the mice [40]. Similar sonication conditions as for the subcutaneous tumor were used (1 MHz; 2 W/cm^2^; 50% duty cycle; 6 min) with plasma collection before and after sonication. Control samples were obtained from mice with tumors not treated with ultrasound. The plasma samples of mice treated with ultrasound showed significant increases in percent change in both protein makers, CEA (7-fold increase, p = 0.048) and CA19-9 (6-fold increase, p = 0.0009) when compared to the untreated control mice (Fig 5A and 5B). The percent change is the comparison between the protein levels pre- and post-sonication. MicroRNAs were not presently studied in this mouse tumor model and will be in the future.

### Patient studies

#### Release of biomarkers from uterine fibroids in patients

**Fig 6:**
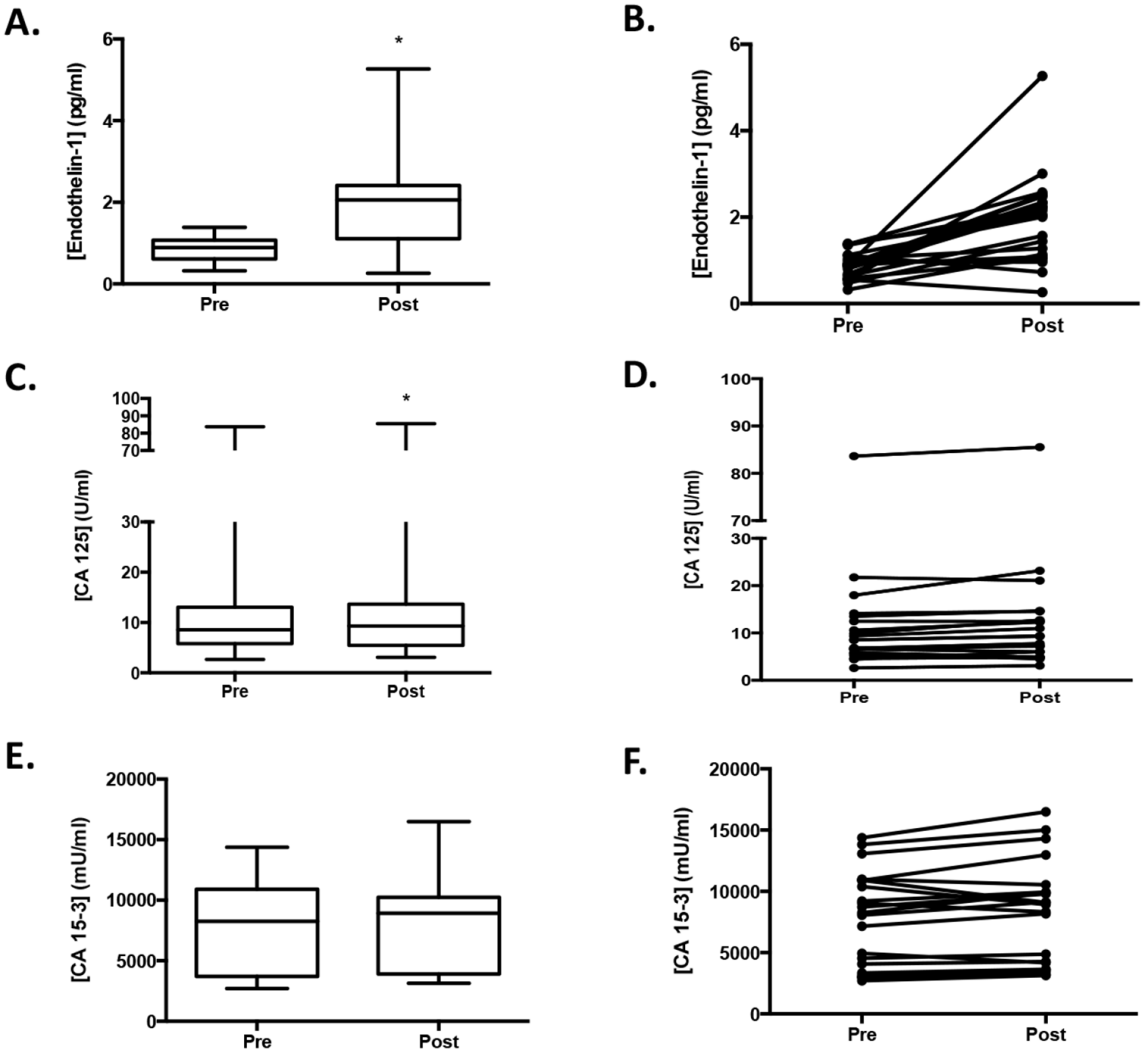
Release of protein-biomarkers from uterine fibroids in patients. The treatment of uterine fibroids with MRg-FUS showed significant (*p≤0.05) increase in release of endothelin-1 (**A**) CA125 (**C**) and a not significant increase in CA15-3 p = 0.06 (**E**), in the post-treatment samples when compared to the pre-treatment plasma samples. The change of pre- versus post-values of each biomarker in individual patients is shown for endothelin-1 (**B**), CA125 (**D**) and CA15-3 (**F**). Box of the plots indicate 25^th^ to 75^th^ percentile with the median and whiskers are maximum to minimum values.

**Fig 7:**
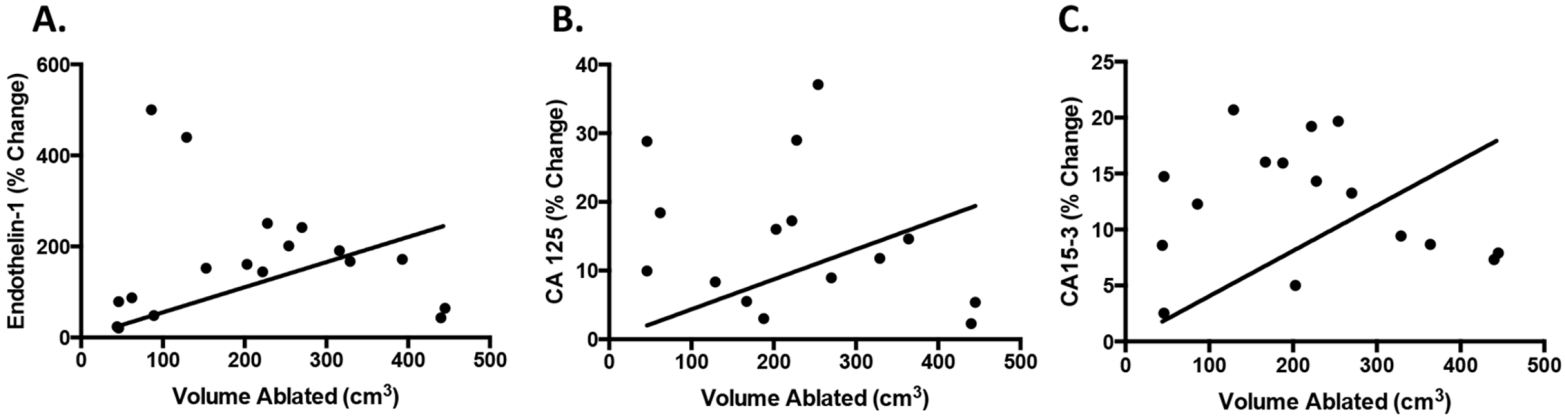
Correlation of biomarker-release to volume of uterine fibroid ablated in patients. The percent change of biomarker in pre- and post-treatment plasma samples was compared to the volume of the fibroid that was ablated and showed a correlation for endothelin-1 p = 0.0026, R^2^ = 0.541 (**A**), CA125 p = 0.0073, R^2^ = 0.129 (**B**) and CA15-3 p = 0.0004, R^2^ = 0.3 (**C**).

To extend our study to a clinical setting, we collected plasma samples from 16 patients who were undergoing routine MRg-FUS treatment of uterine fibroids [7, 8]. Twenty-one samples were obtained in total, as 5 patients had a second treatment of the same fibroid about 7–14 days after initial treatment. The ExAblate 2100 MRgFUS device was operated at 1.1 MHz. Treatments required an average of 116±35 sonications, using an average sonication energy of 3244±840 J delivered over 20 seconds, resulting in a median temperature within the targeted fibroid of 61±4°C. Samples were collected pre- and post-treatment and checked for three protein biomarkers and three miRNA’s. The average volume of the fibroids was 340±169 cm^3^, with ablation volume being 213±131 cm^3^, corresponding to 68±25% of the fibroid volume. Levels of Endothelin-1 (p<0.0001; Fig 6A) and CA125 (p = 0.0175; Fig 6C) significantly increased after treatment, while changes in CA15-3 levels (p = 0.06; Fig 6E) did not reach significance. Individual samples changes with treatment are shown for endothelin-1 (Fig 6B), CA125 (Fig 6D) and CA15-3 (Fig 6F). The smallest fibroid treated was 46 cm^3^ with 100% of the volume ablated and it showed a percent change for all three proteins in plasma pre- and post-treatment (endothelin-1: 78.8%; CA125: 9.95%; CA15-3: 2.54%). Note that samples that did not have a change in pre- and post-treatment values, had detectable pre-treatment values, showing the presence of the biomarkers in the plasma. The percent change of endothelin-1 in the pre- and post-treatment samples showed a linear correlation to the volume of the fibroid ablated (p = 0.0026; R^2^ = 0.541) (Fig 7A), while the other protein biomarkers showed significance of the linear regression trend but showed high variability in the data even if fitted with a quadratic regression (CA125: p = 0.0073, R^2^ = 0.129; CA15-3: p = 0.0004, R^2^ = 0.3) (Fig 7B and 7C).

**Fig 8:**
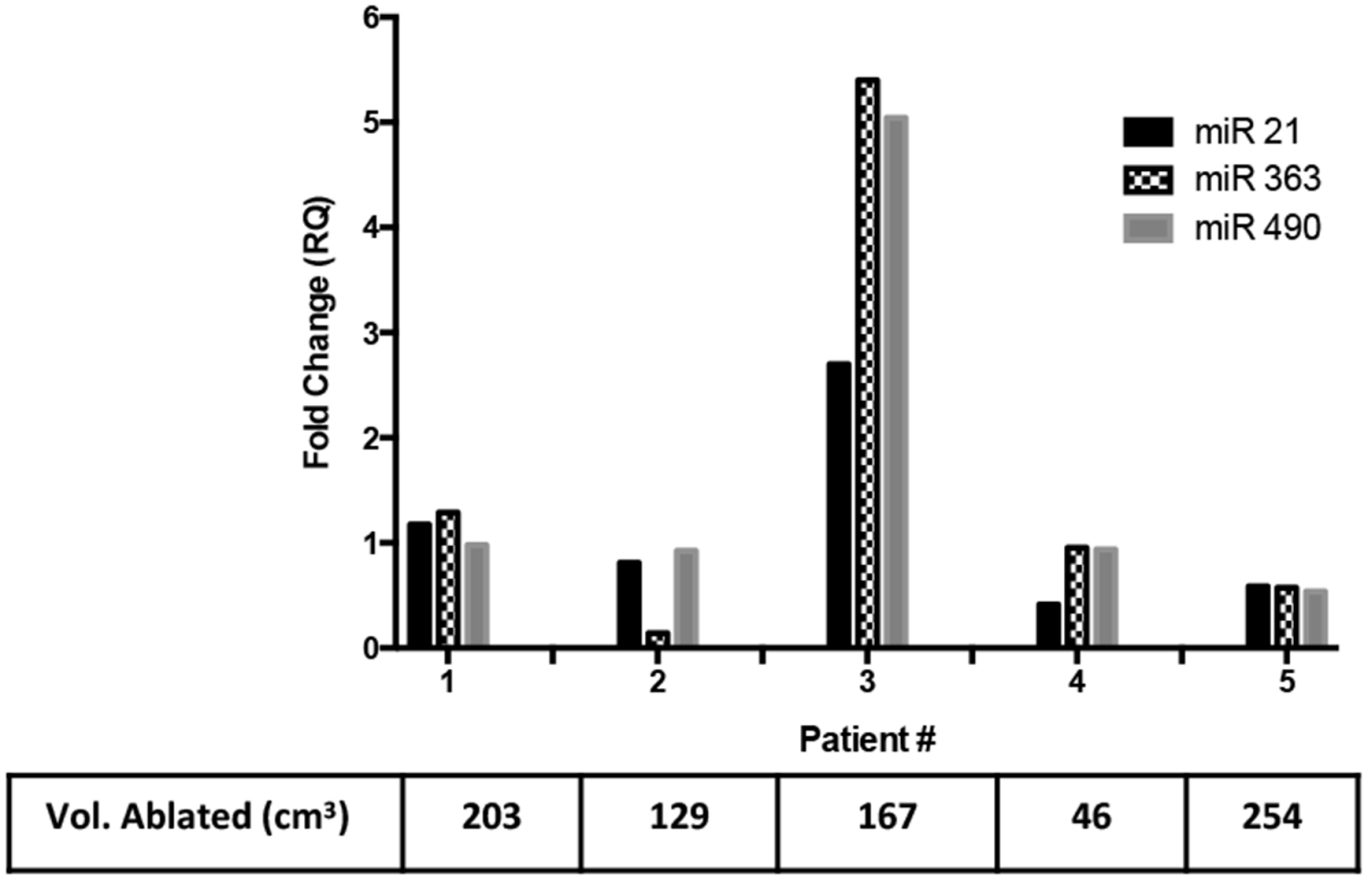
Release of miRNA-biomarkers from uterine fibroid patients. Five patients were tested for an increase in common miRNA found in uterine fibroids. Only one patient (#3) showed fold-increases in the post-treatment samples when compared to pre-treatment samples for miR-21 (2.7-fold), miR-363 (5.4-fold) and miR-490 (5.04-fold). There seemed to not be any correlation of the fold change to the volume of the uterine fibroid ablated (table corresponding to x-axis).

Three miRNAs were also studied in the pre- and post-treatment samples in 5 of the patient samples. The miRNAs chosen are highly expressed in uterine fibroids [41–43]. This was done as a trial to see if miRNAs found in normal uterine fibroids would be good indicators to use or if we would need to do a whole miRNA-panel study. Only one of the 5 patients (#3) showed a 2.7-fold increase in miR-21, a 5.4-fold increase in miR-363 and a 5-fold increase in miR-490 (Fig 8). The other 4 patients did not show any significant increase, which could be due to the absence of the miRNAs in the patient fibroids or a very low concentration that did not allow for the detection of a change. There was no correlation of the miRNA levels to the volume of the ablated fibroid (Fig 8). A larger panel of miRNAs systematically chosen or known to be produced by biopsy-studies of the individual’s fibroid would be required for further results and this was not possible within the scope of the current study.

## Discussion

These results demonstrate the ultrasound-dependent release of multiple biomarkers from cells in culture, in living tumor bearing mice and in humans. This release was seen with multiple biomarkers from a single cell line as well as from multiple cell lines in culture. Protein biomarkers that have different half-lives were shown to be released and detected after sonication in living subjects. The half-life of CEA is known to be 2.5 h with 95% loss within 10 min [37] and CA19-9 is 6 h [38] in nude mice. In humans, the half-life of endothelin-1 has been shown to be between 1 min to 7 h [44], whereas those of CA125 and CA15-3 are shown to be 5–7 days [45, 46]. MicroRNAs are known to be stable in plasma with long half-lives (>24 h *ex vivo*) [31] and an increase in their release with ultrasound proves this strategy to be extremely useful for biomarkers with longer half-lives. Ultrasound-dependent release of miRNAs demonstrates that this technique can be used to release intracellular biomarkers, though further studies would be required to characterize the form, whether in vesicles or protein-bound, in which the miRNAs are released. Our evidence of the use of ultrasound in the release of multiple biomarkers opens avenues for the use of this method to characterize lesions. The variability of biomarkers pre-treatment and percent change levels, both in mice and humans, indicate the usefulness of a panel of biomarkers for characterization. Biomarker panels would allow for an easier and more robust characterization of metastatic as well as primary lesions as is the consensus for the detection and management of disease [15–17, 19, 47].

We also demonstrated the ability of the technique to work with different cell lines. In working with several cell lines, we have observed that a limitation in seeing the spike in released biomarkers in the blood after ultrasound treatment is the sensitivity of the method used to detect the biomarker. The sensitivity of the ELISA is not always enough either to detect the biomarker in media of cells in culture or to detect a difference in the biomarker levels before and after sonication. This limitation is more pronounced in trying to see changes in biomarker levels in the blood of tumor bearing mice. We do expect that the use of more sensitive methods like nanosensors [48–50] and mass spectroscopy [51] for biomarker detection will help to overcome this limitation. It has also been shown that the introduction of microbubbles or nanobubbles into the media of cells in culture combined with sonication at much higher intensities results in a higher release of biomarkers; this could be a method for future exploration [52, 53].

Lesions within the liver are known to be difficult to differentiate from normal tissues by standard ultrasound imaging alone [54]. Ultrasound-dependent release of biomarkers from our liver tumor model suggests a clinical application of this method for potential liver tumor detection and characterization. Our proposed method would allow for a clinically easier method involving the sonication of the entire liver and looking for a change in biomarkers in pre- and post-sonication blood draws. This method of amplification could lead to the earlier detection of liver tumors in the clinic. This provides proof-of-concept that this method could be ultimately used in routine preventive medicine, where a superficial organ that can be easily accessed by ultrasound, like the liver or breast, can be sonicated and blood analyzed for biomarker spikes resulting from the sonication. In the absence of a tumor, an increased release in a tumor specific biomarker would not be observed. This could lead to a much earlier detection method for lesions in these tissues that are otherwise masked by density of the tissue when observed by imaging, the method of preference presently in practice. This study also shows that the two tumor models, subcutaneous and liver, may show differences in biomarkers released. We initially postulated that the liver-tumor model being within an organ and potentially more vascular would allow for a higher amount of biomarker to be released due the sonication and therefore a higher percent change of biomarker released. We observed that this does not hold true for the protein biomarkers studied and conclude that the copy numbers of the biomarkers and their release may be varied contingent on the environment surrounding the tumor that may likely cause these differences. We will further pursue studies in transgenic mice models of cancer where the normal vasculature will allow us to study this in more detail.

LNCaP cells grown subcutaneously in mice show a large number of dilated blood vessels causing the tumors to be poorly differentiated, with extravasation of blood (ecchymosis) leading to hematoma [55, 56]. Release of the biomarkers into the plasma with sonication of these tumors was difficult. Clearance of PSA was not thought to be the issue due to the fact that the half-life of PSA is known to average at 11 h with a rapid clearance of 6.4 h in nude mice [57]. We concluded that in tumors with high blood clots the biomarkers released might not reach the circulation to be detected by a blood test but in fact get trapped in the extracellular matrix. This demonstrates a potential limitation to our method to the ability of the released biomarkers to reach the circulation in tumors that may be of more of a hematoma type. A more systematic study of the kinetics of the release of the biomarkers from such tumor-types is required and will be undertaken in the future.

Future studies will be performed to determine the effectiveness and sensitivity of ultrasound-release of biomarkers in microscopic-tumor determination in comparison to currently used imaging and blood-biomarker based methods. The sensitivity of the ultrasound-release of biomarkers would be governed by a number of variables as discussed above such as tumor/cell type, tumor volume and number of viable tumor cells, the organ involved, the degree of tumor vascularization and the biomarker-panels studied, their abundance, location within or on the cell, detection methods as well as kinetics of release. The present study of using panels of biomarkers, when combined with mathematical modeling could help in quantifying the expected increased sensitivity for ultrasound-release of biomarkers.

The observed plasma biomarker changes in the patients undergoing MRg-FUS gives validation to the use of focused ultrasound to characterize visible lesions. The detection of the increase of biomarker after fibroid ablation shows that the concentration of the released biomarker is substantial enough to be detected despite the large patient blood volume. This could be due to the cellular destruction and increased vascular permeability of the sonication. The percent change for endothelin-1 is much higher than the other two protein biomarkers and we reason this is due to the possibility of varying copy numbers of the biomarkers within the fibroids. We also observed that even though biomarkers were detectable before treatment, we did not always see a post-treatment change. This could be due to either protein denaturation effects of the MRg-FUS or the increase being too small to detect by the methods used. The post collection of blood was within a 30-min window post-treatment for all samples and this may not be the optimum time of release into the blood for all biomarkers. Further optimization of the sonication and interval-collection of blood post sonication together with evolution of more sensitive detection methods would likely help to overcome these issues. This proof that MRg-FUS release of biomarkers works shows the usefulness of this method in a less-invasive characterization of visible lesions that otherwise would need to be biopsied. The ability to evaluate the release of biomarkers from separately targeted tumors may also be an advantage over regular needle biopsies. A more systematic study of the protein and nucleic acid biomarkers found in excised or biopsied fibroid samples will be done to identify biomarkers usable for the MRg-FUS-release. Future studies will include initial biopsies of the fibroid to determine the presence of specific biomarkers before the MRg-FUS, instead of assuming the presence of the biomarker. This would be followed by detection of the release of the identified-biomarkers after MRg-FUS treatment of the fibroid. These studies will allow for us to obtain a distinct panel of biomarkers that can be used for future non-biopsied-MRg-FUS fibroid treatments.

The individualization of this method by the pre-treatment biomarker levels allows every subject to serve as their own internal control and baseline for this method allowing for concise characterization and detection of tumors. Normalization of biomarker levels within a population is a proven limitation in biomarker use and introduction of newly discovered biomarkers in the detection of disease [58]. The variability of the pre-treatment values of biomarkers in the patients shows us the necessity of individual patient normalization. This allows for us to build on our mathematical modeling [59] of tumor size and biomarker release, allowing for better individualized normalization.

One of the clinical applications that this method can be readily used for is the characterization of lesions that are incidentally found while imaging studies are performed for other purposes [60]. MRgFUS can be used either with a cell-pemeabilization or ablative energy deposition and panels of biomarkers from the plasma could help in characterizing the ‘incidentaloma’. In such clinical cases, there may not be any objection to the ablation of the lesion. Panels of biomarkers could be chosen allowing for potential characterization of benign or malignant tumors.

In conclusion, we have proven that our method for the use of ultrasound to amplify and localize the source of biomarkers can be extended to multiple panels of biomarkers and cell types in culture and in living subjects. This technique could prove to be clinically useful in characterization of visible lesions with the detection of biomarker levels in the blood before and after sonication, by continued human studies. Improvement in the sensitivity of blood biomarker detection techniques will further enhance the use of our method for earlier detection of lesions. This method addresses the major limitations of current biomarker studies by increasing the biomarker levels and enabling localization of the biomarker-release site. The ability for the subject to be his or her own internal control to the biomarker levels eliminates a major limitation of biomarker studies of vast variability of background or normal levels in individuals for the detection of disease. Future directions will involve the optimized treatment and characterization of incidentally found lesions and even earlier detection of lesions that are not yet visible by current imaging methods. This method of ultrasound-release of a comprehensive panel of biomarkers with individualized normalization of biomarker levels shows a progressive path for earlier cancer detection.
